# USP11 promotes glycolysis by regulating HIF‐1α stability in hepatocellular carcinoma

**DOI:** 10.1111/jcmm.18017

**Published:** 2024-01-16

**Authors:** Lijun Qiao, Weibin Hu, Linzhi Li, Xin Chen, Liping Liu, Jingbo Wang

**Affiliations:** ^1^ College of Pharmacy, Shenzhen Technology University Shenzhen Guangdong China; ^2^ Department of Hepatobiliary and Pancreas Surgery The Second Clinical Medical College, Jinan University (Shenzhen People's Hospital) Shenzhen Guangdong China; ^3^ Department of Hepatobiliary and Pancreas Surgery, The First Affiliated Hospital Southern University of Science and Technology Shenzhen Guangdong China; ^4^ Institute for Brain Research and Rehabilitation, South China Normal University Guangzhou Guangdong China; ^5^ Laboratory of Molecular Immunology, State Key Laboratory of Genetic Engineering, School of Life Sciences Fudan University Shanghai China

**Keywords:** deubiquitinase ubiquitin‐specific protease 11, glycolysis, hepatocellular carcinoma, hypoxia‐inducible transcription factor‐1α, tumor progression

## Abstract

Understanding the mechanisms underlying metastasis in hepatocellular carcinoma (HCC) is crucial for developing new therapies against this fatal disease. Deubiquitinase ubiquitin‐specific protease 11 (USP11) belongs to the deubiquitinating family and has previously been reported to play a critical role in cancer pathogenesis. Although it has been established that USP11 can facilitate the metastasis and proliferation ability of HCC, the underlying regulatory mechanisms are poorly understood. The primary objective of this research was to reveal hitherto undocumented functions of USP11 during HCC progression, especially those related to metabolism. Under hypoxic conditions, USP11 was found to significantly impact the glycolysis of HCC cells, as demonstrated through various techniques, including RNA‐Seq, migration and colony formation assays, EdU and co‐immunoprecipitation. Interestingly, we found that USP11 interacted with the HIF‐1α complex and maintained HIF‐1α protein stability by removing ubiquitin. Moreover, USP11/HIF‐1α could promote glycolysis through the PDK1 and LDHA pathways. In general, our results demonstrate that USP11 promotes HCC proliferation and metastasis through HIF‐1α/LDHA‐induced glycolysis, providing new insights and the experimental basis for developing new treatments for this patient population.

## 1 INTRODUCTION

Hepatocellular carcinoma (HCC) is a serious type of primary liver cancer that has a major influence on cancer‐related morbidity and mortality worldwide.^1^, ^2^ In the past few years, the prevalence of HCC rapidly increased as a result of hepatitis infection and alcohol and nonalcoholic steatohepatitis (NASH) cirrhosis.^3^ HCC is one of the highly aggressive solid tumours characterized by evasion of apoptosis, aberrant angiogenesis and an abnormal cell cycle.^4^ Due to poor prognosis, HCC always be diagnosed at an advanced stage, which limited the effective curative interventions such as percutaneous local ablation and surgical resection.^5^ Systemic therapy with multikinase inhibitors, only yields minor survival benefits, which are always counteracted by considerable side effects.^6^ Immune checkpoint inhibitors are potential novel treatment for HCC that have improved patient survival when combined with targeted therapies; however, the response rate is under 50%.^6^ Therefore, elucidation of the mechanisms underlying HCC initiation, progression and metastasis is important for seeking additional therapeutic procedures to prolong the overall survival of HCC patients.

Hypoxia is proved important role in multi‐cellular biological functions including proliferation, angiogenesis, metastasis and glycolysis, which related to some solid cancers, including HCC. The oxygen‐sensitive component known as the hypoxia‐inducible transcription factor‐1α (HIF‐1α) plays critical roles in response to hypoxia. According to reports, substantial expression of HIF‐1α in HCC is associated with patients' poor clinical prognosis.^7^ Moreover, high HIF‐1α expression accounts for drug resistance in HCC.^8^, ^9^ In addition, mounting evidence suggests that HIF‐1α can increase glycolysis in cancer by upregulating the expression of downstream targets such as PDK1 and LDHA, which are glycolytic‐related genes.^10^, ^11^ It has been documented that tumour cells with a high glycolytic rate accelerate chemotherapy resistance.^12^ Recent studies have outlined the potential benefits of inhibiting tumour glycolysis to increase its treatment sensitivity.^13^

Ubiquitin‐specific protease 11 (USP11) is a member of the deubiquitinating enzyme family, which is located in the cell nucleus.^14^ It has been established that USP11 participates in cancer development. A non‐biased screen of 67 human deubiquitinases revealed that USP11 is the most decisive determinant that leads to tumour cell initiation and progression.^15^ It has been demonstrated that USP11 promotes colorectal cancer and HCC growth and metastasis by stabilizing NF90 and activating the ERK/MAPK signalling pathway.^16^ Although USP11 plays a critical role in HCC progression, and our earlier research established that USP11 inhibits autophagy through targeting ERK/mTOR pathway to promote HCC metastasis,^16^ the mechanisms of USP11 in HCC glycolysis remain poorly understood.

Various biological processes, including hypoxia, are related to protein homeostasis, which maintained by post‐translational modification. The two major kinds of post‐translational modification are ubiquitination and deubiquitination.^17^ HIF‐1α proteins are regulated by ubiquitylation and proteasomal degradation. In an oxygen‐dependent way, pVHL, an E3 ligase, interacts directly with HIF‐1α for ubiquitin‐mediated degradation.^18^ It has been reported that the deubiquitylase ovarian tumour domain containing 6B (OTUD6B) reduces HIF‐1α accumulation by deubiquitinating and stabilizing pVHL in HCC cells under hypoxia, then suppressing HCC metastasis.^19^ Moreover, current evidence suggests that USP22 could promote hypoxia‐induced HCC glycolysis and stemness by deubiquitinating and stabilizing HIF‐1α.^7^ Though some USPs targeting HIF‐1α have been identified, there is a need to investigate USPs that are involved in hypoxia.

In the current study, we found that USP11 could interact with HIF‐1α and maintain its protein stability by removing ubiquitin. HIF‐1α actively participated in glycolysis and promoted tumour cell proliferation under hypoxia conditions. We also demonstrated that USP11 could enhance glycolysis through PDK1 and LDHA‐related mechanisms. Overall, our findings point to USP11's crucial function in HCC development, which offers fresh information for the future treatment of liver cancer.

## 2 MATERIALS AND METHODS

### 2.1 Cells

PLC/PRF/5, Hep3B, HEK 293T and Huh7 cells were cultured in Dulbecco's modified Eagle Medium (DMEM) containing 10% fetal bovine serum (FBS). All cells were cultured with penicillin and streptomycin at 37°C and 5% CO_2_. Cocl2 was added to 20 μM to oxygenate the cells, and MG132 was used to inhibit proteasome activity.

### 2.2 siRNAs, plasmids and transfection

Cells were seeded and incubated in an antibiotics‐free medium for 24 h. Chemically modified Stealth small interfering RNAs (siRNAs) targeting USP11 and HIF‐1α and control siRNAs were purchased from Ribobio (Ribobio, China). The Flag‐USP11, Flag‐HIF‐1α, Flag‐EGLN1, Flag‐EGLN2, Flag‐EGLN3 and Flag‐VHL plasmids encoded the N‐terminally Flag‐tagged proteins. The HA‐USP11 plasmid encoded an N‐terminally HA‐tagged USP11 protein. Next, siRNAs and plasmids were transfected into the cells using Lipofectamine 3000 (L3000008, Invitrogen, Life Technologies, CA, USA) according to the manufacturer's instructions. The siRNA sequences were as follows:

siUSP11‐1: 5′‐ACCGATTCTATTGGCCTAGTA‐3′;
siUSP11‐2: 5′‐CTGCGTCGGGTACGTGATGAA‐3′;
siHIF‐1α: 5′‐GTGGATAGCGATATGGTCATT‐3′.

Forty‐eight hours after siRNA transfection, cells were collected for further analysis.

### 2.3 RNA extraction and real‐time quantitative PCR (RT‐qPCR)

RNA was extracted with a TRIzol reagent (15596026, Invitrogen, USA). cDNA was synthesized using random primers with the PrimeScript™ RT Reagent Kit with gDNA Eraser (RR037A, Takara, Japan). After reverse transcription, qPCR was performed to evaluate the expression levels of indicated gene using SYBR® Premix ExTaq™ (RR420A, Takara, Japan) and monitored on a DNA Engine Peltier thermal cycler (ABI 7300, USA). Cycling conditions were: initial denaturation at 95°C for 3 min, followed by 40 cycles of 95°C for 15 s and 60°C for 30 s. Primer sequences were as follows:

USP11 forward, 5′‐TATAAGCAGTGGGAGGCATACG‐3′;
USP11 reverse, 5′‐ATGACCTTGCGTTCAATGGGT‐3′;
HIF‐1α forward, 5′‐GAACGTCGAAAAGAAAAGTCTCG‐3′;
HIF‐1α reverse, 5′‐CCTTATCAAGATGCGAACTCACA‐3′;
PDK1 forward, 5′‐GAGAGCCACTATGGAACACCA‐3′;
PDK1 reverse, 5′‐GGAGGTCTCAACACGAGGT‐3′;
LDHA forward, 5′‐ATGGCAACTCTAAAGGATCAGC‐3′;
LDHA reverse, 5′‐CCAACCCCAACAACTGTAATCT‐3′

### 2.4 RNA‐Seq

Samples from three biologically separate experiments were collected. The NEBNext Ultra RNA Library Prep Kit for Illumina (E7530, New England Biolabs, MA, USA) was used to create the RNA‐Seq libraries, and the Agilent 2100 Bioanalyzer (Agilent Technologies) was used to validate them. On a NovaSeq 6000 sequencer, libraries were sequenced. Using HISAT2 (version 2.0.5), the clean reads were aligned to the human (hg37) genomes. The featureCounts (version 1.5.0‐p1)‐calculated fragments per kilobase per million fragments mapped (FPKM) was used to determine the differentially expressed genes (DEGs) between groups. Using edgeR (version 3.24.1), the *p*‐value was adjusted to account for multiple testing. Using the R software's limma tool, the DEGs were found using the cut‐off criterion. *p* 0.05 and |log2 fold change| > 2.0 were the cut‐off values for the upregulated or downregulated genes.

### 2.5 Colony formation assay

1–2 × 10^3^ cells were seeded in a six‐well plate for 10–14 days. Cells were washed with PBS and fixed with methanol for 15 min followed by 30 min's crystal violet staining. Finally, the colonies were counted and captured.

### 2.6 Western blot

Total proteins were extracted with radioimmunoprecipitation assay (RIPA) lysis buffer with protease inhibitor (11697498001, Sigma, USA) and quantified (P0012, Beyotime, China). Before being transferred to PVDF membranes, lysates were resolved on 10% SDS/PAGE gels. Specific antibodies against USP11 (22340‐1‐AP, Proteintech, China), HIF‐1α (#3716, CST, USA), PDK1 (sc‐515944, Santa Cruz, USA), LDHA (sc‐137243, Santa Cruz, USA), MYC (AB0001), HA (AB0004), Flag (AB0008) (all from Abways, China), actin (20536‐1‐AP, Proteintech, China) and GAPDH (HC301‐01, Transgene, China) were used for the western blotting study. Protein levels were evaluated by scanning the intensities of the bands by ImageJ software (NIH, Bethesda, MD, USA). and each band were normalized to the intensity of actin in the same field. Finally, the relative intensity of each band was calculated based on the intensity of NC and each experimental group using GraphPad Prism (GraphPad Software, Inc. La Jolla, USA).

### 2.7 Co‐immunoprecipitation (Co‐IP)

Two hundred ninety‐three T cells were transfected and incubated for 48 h for transient transfection and co‐immunoprecipitation experiments. After that, lysis buffer was used to lyse the cells. For each immunoprecipitation, 0.4 mL of lysate was treated overnight at 4°C with 4 μL of the specified antibody or control IgG. After 2 h, 20 μL of magnetic beads (M8823, Millipore, USA) were introduced. The precipitates were ultimately examined using a conventional western blot. ECL detection tools were used to see protein bands.

### 2.8 Luciferase assay

The hypoxia response element (HRE)‐Luciferase, a pGL2 vector containing three hypoxia response elements, was purchased from Addgene (26731). Renilla luciferase‐expressing vector pGL4.74‐Rluc was obtained from Promega (Madison, WI, USA). Cells were cotransfected with the vector or USP11 (HIF‐1α) and NC or siHIF‐1α (siUSP11). After being cultured for 48 h, cells were harvested for luciferase assays by the Dual‐Luciferase Reporter Assay System (Promega, USA).

### 2.9 Immunofluorescence

At 10^5^ cells per well, Hep3B cells were divided among 12‐well plates. Cells were washed and fixed for 15 min after being transfected for 48 h. PBS with 0.5% Triton X‐100 was used to further permeabilize the cells for 15 min. After that, the cells were twice rinsed, blocked in 5% w/v BSA/PBS for 30 min and then incubated overnight with the primary antibody. After being washed, the cells were exposed for 60 min to a secondary antibody (A‐11012, Alex Fluor® 594, 1:500; A‐11001, Alex Fluor® 488, 1:500). DAPI was used as a counterstain for the cell nucleus. The cells were cleaned before being photographed using a confocal laser by Leica (Germany).

### 2.10 Migration assay

1 × 10^4^ HCC cells were washed and suspended with serum‐free medium and added to the upper layer of transwell plates (Corning, USA). 0.5 mL DMEM (with 20% FBS) was added to the lower chambers. After the indicated time, the filter was fixed with methanol for 10 min before being stained for 15 min. Cells on the upper membrane were gently removed. Migrated cells on the lower membrane were captured under a microscope at 100 × magnification and counted. Each experiment was repeated at least three times.

### 2.11 Lactate content measurement

Intracellular lactate contents of Hep3B and Huh7 cells were determined using the L‐Lactate Assay Kit (BC2235, Suolaibao, China). Hep3B and Huh7 cells were cultured in DMEM basic medium with 10% FBS under normoxic or hypoxic conditions. The cells and medium were collected for lactate content measurement according to the manufacturer's instructions.

### 2.12 Immunohistochemistry (IHC)

The expression level of USP11 and HIF‐1α was assessed in paracancerous tissues and HCC tissues as described previously.^20^ Tissues were fixed in 4% paraformaldehyde, embedded in paraffin and cut into sections for immunohistochemical staining. Immunohistochemistry staining analysis was performed as described.^20^ In case of disagreements between the investigators, the data for the slide were discarded. This study was approved by the Ethics Committee of Shenzhen Technology University and the Second Clinical Medical College, Jinan University (Shenzhen People's Hospital), and written consent was obtained from all patients.

### 2.13 Statistical analysis

All statistical analyses were performed using SPSS 17.0 (SPSS, Chicago, IL, USA) and GraphPad Prism (GraphPad Software, Inc. La Jolla, USA). Data were presented as the means and Standard Error of Mean (SEM). The Student's *t*‐test was performed, and *p*‐values <0.05 were statistically significant.

## 3 RESULTS

### 3.1 USP11 regulates HIF‐1α protein expression

**FIGURE 1:**
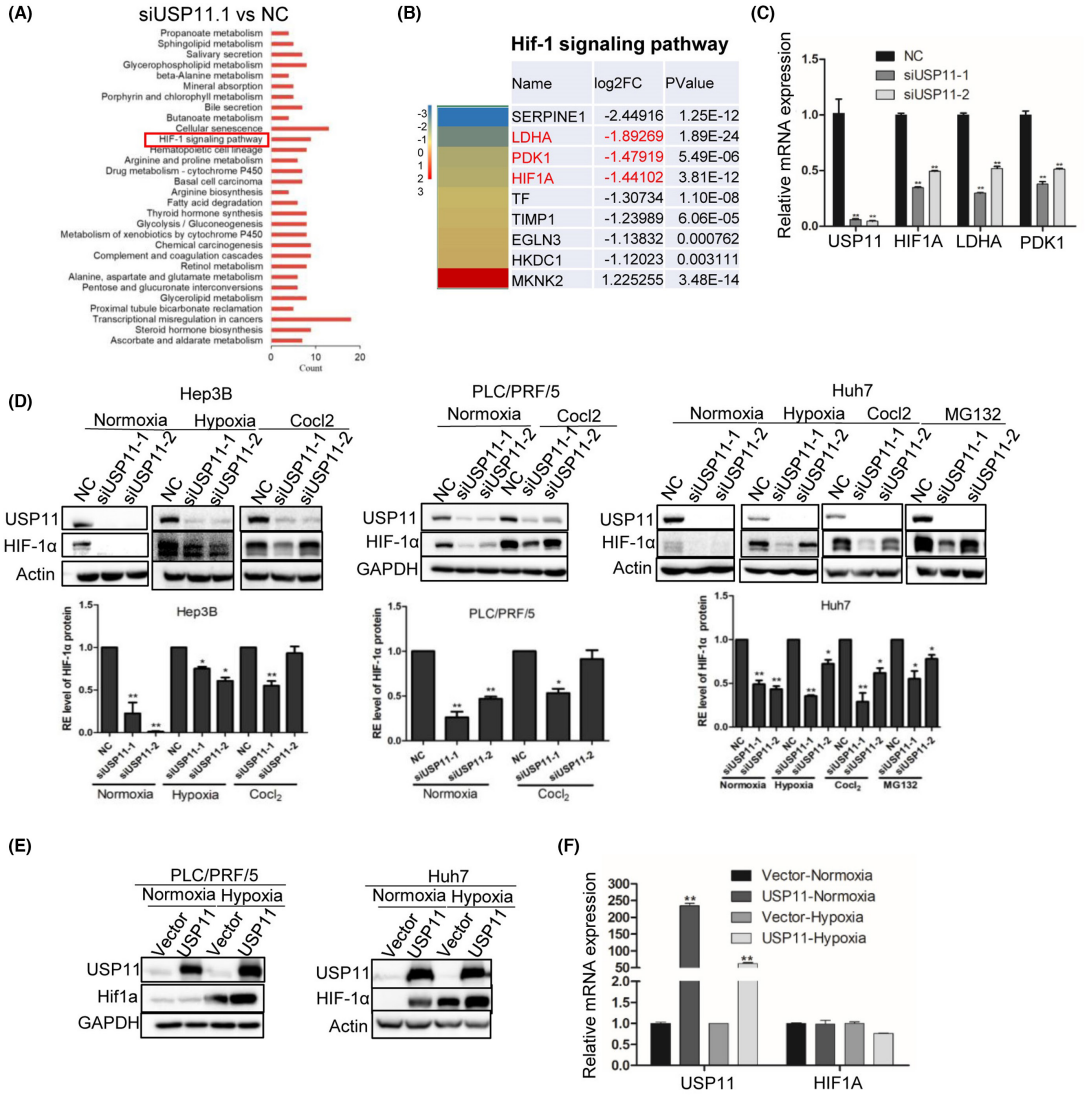
HIF‐1α is the downstream target of USP11 in HCC cells. (A) Multiple KEGG sets were significantly enriched after USP11 silencing in Hep3B cell line compared with control cells based on RNA‐Seq data (cells from *n* = 3 independently grown cultures). (B, C) Expression level of HIF‐1α, LDHA and PDK1 was significantly reduced after USP11 silence. (D) HIF‐1α level was infected by USP11 under normoxia and hypoxia conditions in Hep3B, PLC/PRF/5 and Huh7 cell lines. Cells were cultured under hypoxia (1% O_2_) or normoxia (20% O_2_), 20 μM CoCl_2_ and 10 μM MG132 for 12–48 h and western blot was used to analyse the expression level of USP11 and HIF‐1α. (E) Enhanced expression of HIF‐1α after USP11 overexpression. (F) Expression mRNA level of HIF‐1α was not changed after USP11 overexpression under normoxia and hypoxia conditions. Data are the means ± SEM, *n* = 3 independent experiments; two‐tailed *t*‐tests, **p < 0.01.

To explore the potential roles of USP11 during HCC progression, RNA‐Seq analysis was carried out to identify gene expression changes in Hep3B cells after being transfected with siUSP11. The DEGs were identified (Table S1) and underwent functional enrichment analysis. During the Kyoto Encyclopedia of Genes and Genomes (KEGG) analysis, the most significantly enriched pathways included transcriptional misregulation in cancers, thyroid hormone synthesis, glycolysis and so on (Figure 1A and Table S2). Among them, the hypoxia‐inducible factor‐1 (HIF‐1) signalling pathway was significantly inhibited in siUSP11 cells compared with NC (Figure 1B and Table S3). It is well established that HIF‐1 acts as an oxygen sensor and influences the survival of stressed cells. HIF proteins undergo degradation in normoxic settings but stabilized in low oxygen environments, where they bind to co‐factors and translocate to the nucleus to regulate gene transcription.^21^ The mRNA levels of critical components in the HIF‐1 signalling pathway were also examined, and the results showed that following USP11 knockdown, the mRNA levels of HIF‐1, LDHA and PDK1 were considerably reduced (Figure 1C). HIF‐1α is the key component of the HIF‐1 signalling pathway and has been shown to be critical in cell glycolysis as well as immune regulation.^22^

Reports have shown that HIF‐1 can regulate pyruvate dehydrogenase kinases (PDKs) under hypoxia, preventing tricarboxylic acid cycle and enhancing lactate production in quiescent HSCs.^23^ Therefore, we evaluate HIF‐1α protein expression in HCC cell lines after USP11 knockdown or overexpression under both normoxic and hypoxic conditions. Specifically, the protein level of HIF‐1 was decreased after siUSP11 transfection (Figure 1D) but increased with USP11 overexpression (Figure 1E). Furthermore, the down‐regulation of HIF‐1 protein expression by siUSP11 was partially counteracted by the proteasome inhibitor MG132 (Figure 1D). However, after USP11 overexpression, the mRNA level of HIF‐1 remained steady (Figure 1F), indicating that USP11 may primarily effect HIF‐1α at the translational level, specifically via the proteasome system.

### 3.2 USP11 interacts with and stabilizes HIF‐1α in HCC cells

**FIGURE 2:**
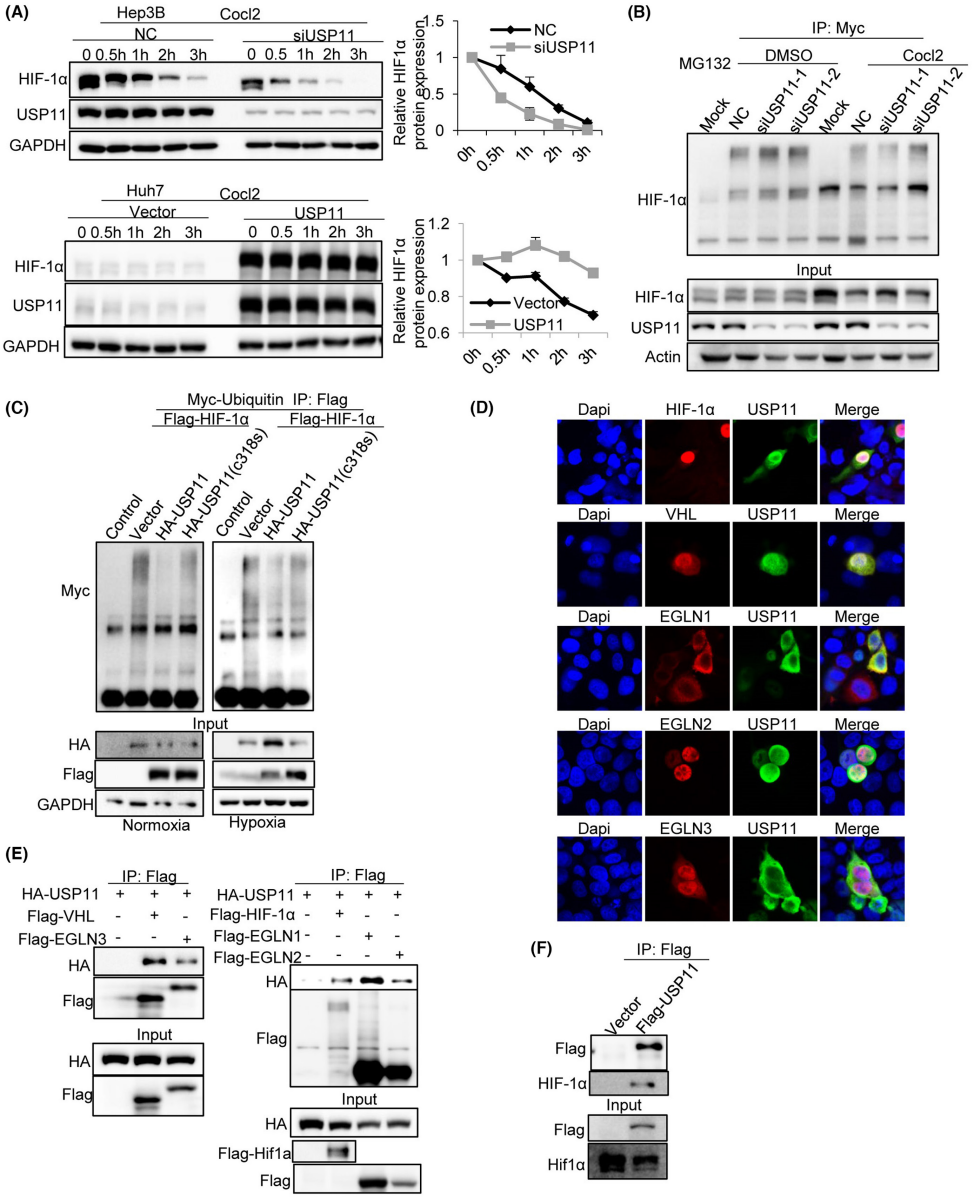
USP11 interacts with HIF‐1α complex and stabilizes HIF‐1α in HCC cells. (A) Hep3B cells were transfected with siUSP11 or USP11 before incubated with Cocl2, the expression level of USP11 and HIF‐1α was detected using western blot at indicated time points. (B) Hep3B cells were transfected with siUSP11 and cultured with MG132. Total cell lysates were subjected to immunoprecipitation assay with MYC beads. (C) HEK293T cells were transfected with plasmids expressing either Flag‐HIF‐1α or HA‐USP11 and Myc‐ubiquitin plasmids. Antibodies against Flag or HA were checked in the precipitations. (D) The subcellular localization of USP11 and HIF‐1α, VHL, EGLN1, EGLN2 and EGLN3 in Hep3B was visualized. Cell nucleus was counterstained with DAPI. (E) HEK293T cells were transfected with plasmids expressing HA‐USP11 and Flag‐VHL or EGLN3, HIF‐1α. Antibodies against Flag or HA were checked in the precipitations. (F) Hep3B cells were transfected with plasmids expressing Flag‐USP11 and western blot analysis was used to detect HIF‐1α.

Given that USP11 is involved in protein deubiquitination, we hypothesized that USP11 could help to stabilize HIF‐1α in HCC cells. The protein synthesis inhibitor cycloheximide (CHX) was applied to cells, and the stability of the HIF‐1α protein was tested under USP11 knockdown and overexpression conditions. After USP11 knockdown in PLC/PRC/5 cells, the HIF‐1α protein degraded more quickly, indicating that HIF‐1α degradation is accelerated in hypoxic environments. Additionally, HIF‐1α expression was markedly upregulated by USP11 overexpression in Huh7 cells (Figure 2A). These findings revealed that USP11 regulated HIF‐1α expression at the post‐translational level. The ubiquitination assay further substantiated that USP11 knockdown significantly increased poly‐ubiquitination of HIF‐1α under normoxic and hypoxic conditions (Figure 2B). Conversely, the poly‐ubiquitination levels of HIF‐1α decreased after USP11 overexpression under normoxic and hypoxic conditions (Figure 2C). To further explore the role of USP11 during HIF‐1α ubiquitination, we introduced a mutation from 318C to 318S in the active site of USP11 to interrupt the catalytic core^24^ and assessed whether the mutation site would affect the ubiquitination level of HIF‐1α. As shown in Figure 2C, compared with HA‐USP11, transfected cells with HA‐USP11 (C318S) increased the poly‐ubiquitination levels of HIF‐1α. These results demonstrated that USP11 affects the stability of the HIF‐1α protein.

To further reveal the potential interaction between USP11 and HIF‐1α, a Co‐IP assay was performed. Then, we cotransfected HA‐USP11 and Flag‐HIF‐1α plasmids into 293T cells. HA‐tagged USP11 protein was incubated with total cell lysate, and the binding affinity of the protein was assessed using western blot analysis. Our results suggested Flag‐HIF‐1α was significantly enriched in USP11 transfected cell lysates (Figure 2E).

It is well established that the E3 ligase, a protein complex formed with VHL and EGLN (prolyl hydroxylases, PHD) family members, serves as an upstream signal of HIFα. Under normoxic conditions, the HIF1α subunit is usually prolyl‐hydroxylated by VHL and EGLN family members, leading to rapid degradation.^25^, ^26^ The co‐localization of USP11 and HIF‐1α or VHL, EGLN1 (PHD2), EGLN2 (PHD1) and EGLN3 were also evaluated in our study. We found that USP11 and EGLN2 are mainly located in the cell nucleus, EGLN1 was localized in the cytoplasm, while VHL and EGLN3 were distributed all around the cell (Figure 2D), consistent with previous studies. Furthermore, the Co‐IP assay confirmed that USP11 could interact with HIF‐1α, VHL, EGLN1, EGLN2 and EGLN3 complex (Figure 2E). Moreover, transfection of Flag‐USP11 could pull the endogenous HIF‐1α down in HCC cells (Figure 2F). These results indicated that USP11 interacts with HIF‐1α and stabilizes it.

### 3.3 HIF‐1α mediates the stimulatory effect of USP11 on HCC cell proliferation and migration in hypoxia

**FIGURE 3:**
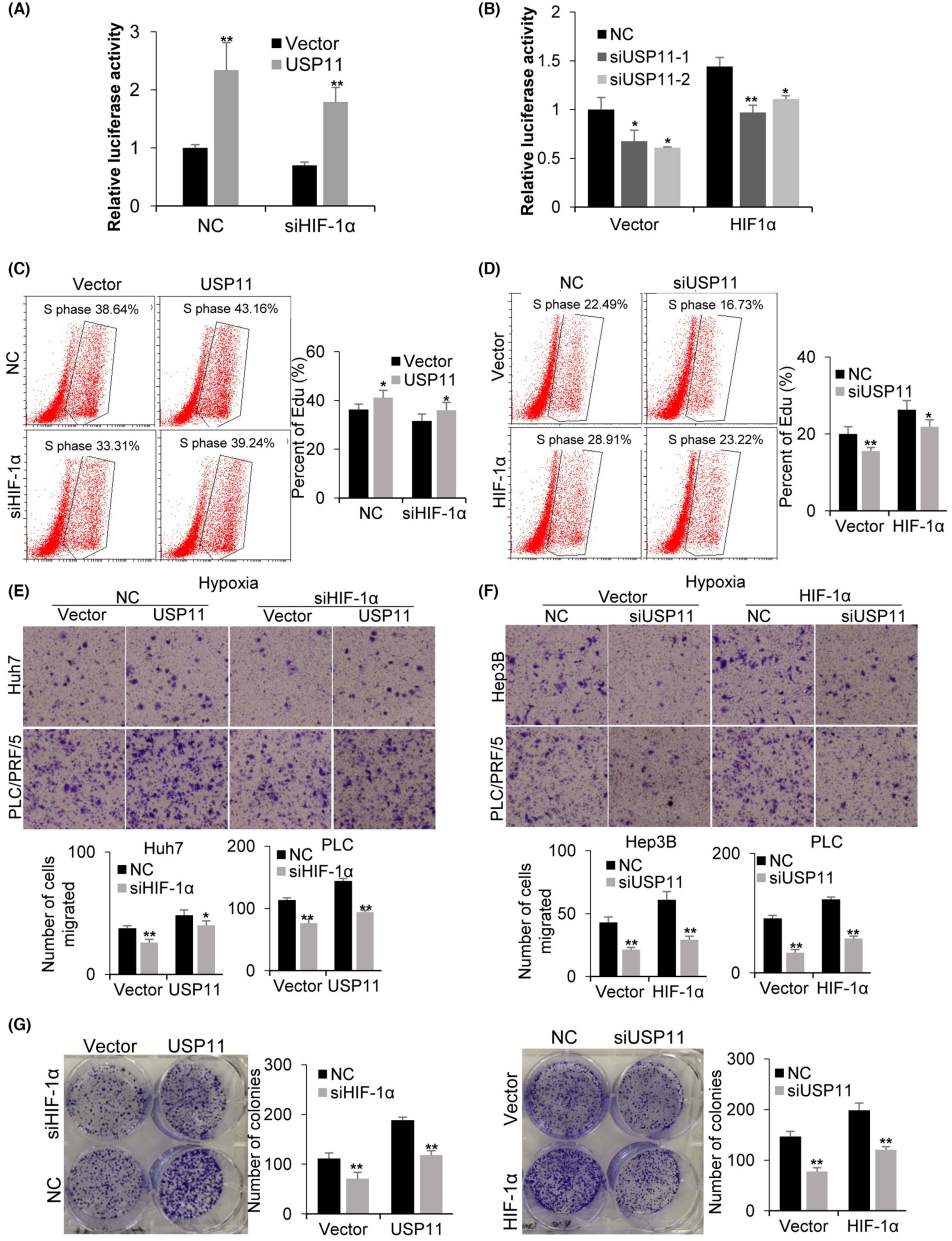
HIF‐1α mediates the promotion effect of USP11 on HCC cell proliferation and migration in hypoxia. Dual‐luciferase reporter assays were performed by co‐transfection of the USP11 and siHIF‐1α (A) or siUSP11 and HIF‐1α (B). EdU assay analysis of the proliferation of HCC cells transfected with USP11 and siHIF‐1α (C) or siUSP11 and HIF‐1α (D). Transwell migration assay of USP11 and siHIF‐1α (E), siUSP11 and HIF‐1α (F) transfected HCC cells. (G) Colony formation assay of USP11 and siHIF‐1α or siUSP11 and HIF‐1α transfected HCC cells. Data are the means ± SEM, *n* = 3 independent experiments; two‐tailed *t*‐tests, *p < 0.05, **p < 0.01.

HIF‐1α‐dependent luciferase (HRE‐Luciferase) experiments were performed as described previously^27^ to confirm whether USP11 could regulate HIF‐1α activity and the expression of HIF‐1α inducible genes. Under hypoxic (1% O_2_) conditions, the transcriptional activity of HIF‐1α remained stable with HIF‐1α knockdown and exhibited a significant increase with USP11 overexpression (Figure 3A). Meanwhile, the luciferase activity decreased markedly with USP11 knockdown (Figure 3B). The transcriptional activity of HIF‐1α was further investigated by examining the induction of HIF‐1α target genes, PDK1 and LDHA with real‐time quantitative PCR. USP11 knockdown cells exposed to normoxia and hypoxia exhibited a significant decrease in PDK1 and LDHA levels in comparison with the NC group (Figure 1C). These findings confirmed that USP11 can modulate HIF‐1α activity.

In previous work, we reported that USP11 might increase both HCC cell migration and proliferation,^16^ and HIF‐1α has been shown to trigger the transcription of genes involved in HCC proliferation, angiogenesis, metastasis and invasion.^28^ Accordingly, we hypothesized that HIF‐1α could mediate the beneficial effect of USP11 on HCC cell proliferation and migration. The influence of USP11 and HIF‐1α on HCC cells was investigated through cell proliferation, migration and colony formation experiments. The EdU assay results demonstrated that the proliferation ability of HCC cells increased after USP11 overexpression in both NC and siHIF‐1α transfected groups (Figure 3C). Correspondingly, USP11 knockdown reduced cell proliferation, and HIF‐1α overexpression restored cell proliferation ability to a certain extent (Figure 3D). The transwell assay revealed that HCC cells’ migratory ability was significantly enhanced after USP11 overexpression, while siHIF‐1α transfection reduced their cell migration ability (Figure 3E). Besides, USP11 knockdown could reduce the cell migration ability, and HIF‐1α overexpression rescued the cell migration ability to a certain extent (Figure 3F). We observed a similar tendency in colony formation assay (Figure 3G). These findings suggested that HIF‐1α may behave as a functional downstream target of USP11 in HCC under hypoxic conditions.

### 3.4 USP11 affects glycolysis by HIF‐1α/LDHA pathway

**FIGURE 4:**
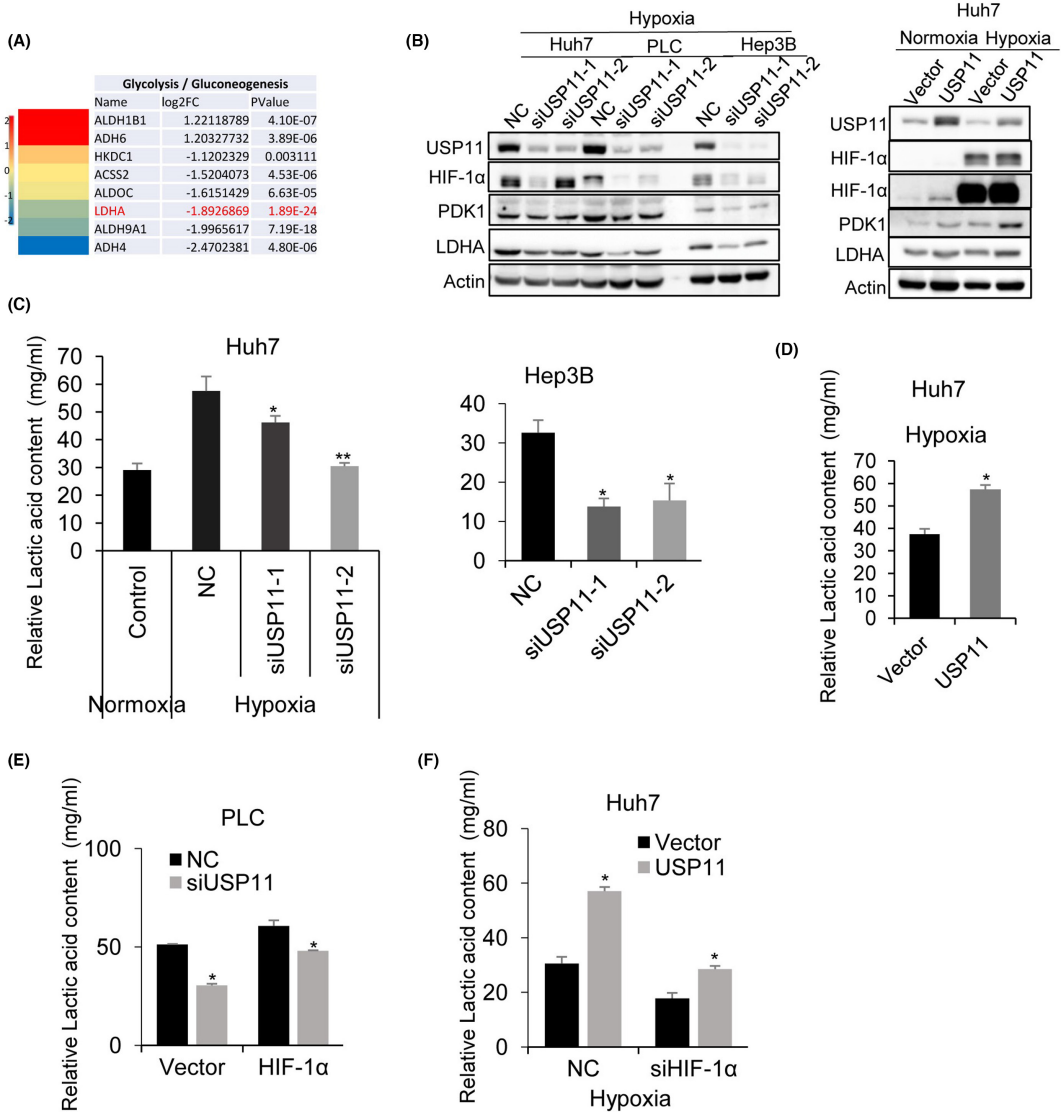
USP11 effects glycolysis by HIF‐1α/LDHA/ pathway. (A) Expression level of LDHA in glycolysis/gluconeogenesis pathway was significantly reduced after USP11 silence based on RNA‐Seq data (cells from *n* = 3 independently grown cultures). (B) Western blot analysis of the effect of USP11 knockdown under normoxia or hypoxia stimulation on HIF‐1α/LDHA pathway. Relative content of lactic acid in siUSP11 (C) or USP11 transfected HCC cells (D). Lactic acid level in HCC cells transfected with siUSP11 and HIF‐1α (E) or USP11 and siHIF‐1α (F).

Given that HIF‐1α plays a critical role in glycolysis,^29^ the influence of USP11 on HCC cell glycolysis was evaluated. RNA‐Seq analysis of siUSP11 and NC transfected HCC cells showed that lactate dehydrogenase A (LDHA) expression was the most significantly downregulated gene in glycolysis pathways (Figure 4A). Higher LDHA expression has been found to be positively connected with malignant progression in many cancers such as gastric, gallbladder, pancreatic cancer, nasopharyngeal cancer and HCC.^30^ LDHA is a protein that is essential during glycolysis, which metabolizes pyruvate to lactate.^31^ The distribution of NADH and FADH2 to the electron‐transport chain is lowered with the help of pyruvate dehydrogenase kinase 1 (PDK1), which inactivates PDH, the mitochondrial enzyme that converts pyruvate to acetyl‐CoA, thereby facilitating glycolysis.^32^ To evaluate whether USP11 could influence the HIF‐1α/LDHA pathway, the expression of HIF‐1α, PDK1 and LDHA protein was quantified after USP11 knockdown in HCC cells. We found a significant decrease in their expression in siUSP11 transfected cells (Figure 4B). To further confirm this, the lactic acid level was quantified. As shown in Figure 4C, the relative lactic acid level in HCC cells was significantly reduced after USP11 knockdown and increased with USP11 overexpression (Figure 4D). HIF‐1α overexpression rescued the decrease in lactic acid level after siUSP11 transfection (Figure 4E). In contrast, USP11 overexpression restored the lactic acid level to a certain extent following HIF‐1α knockdown (Figure 4F). Collectively, our results demonstrated that USP11 could regulate glycolysis of HCC cells in a HIF‐1α//LDHA‐dependent manner.

### 3.5 USP11 expression is positively correlated with HIF‐1α in HCC

**FIGURE 5:**
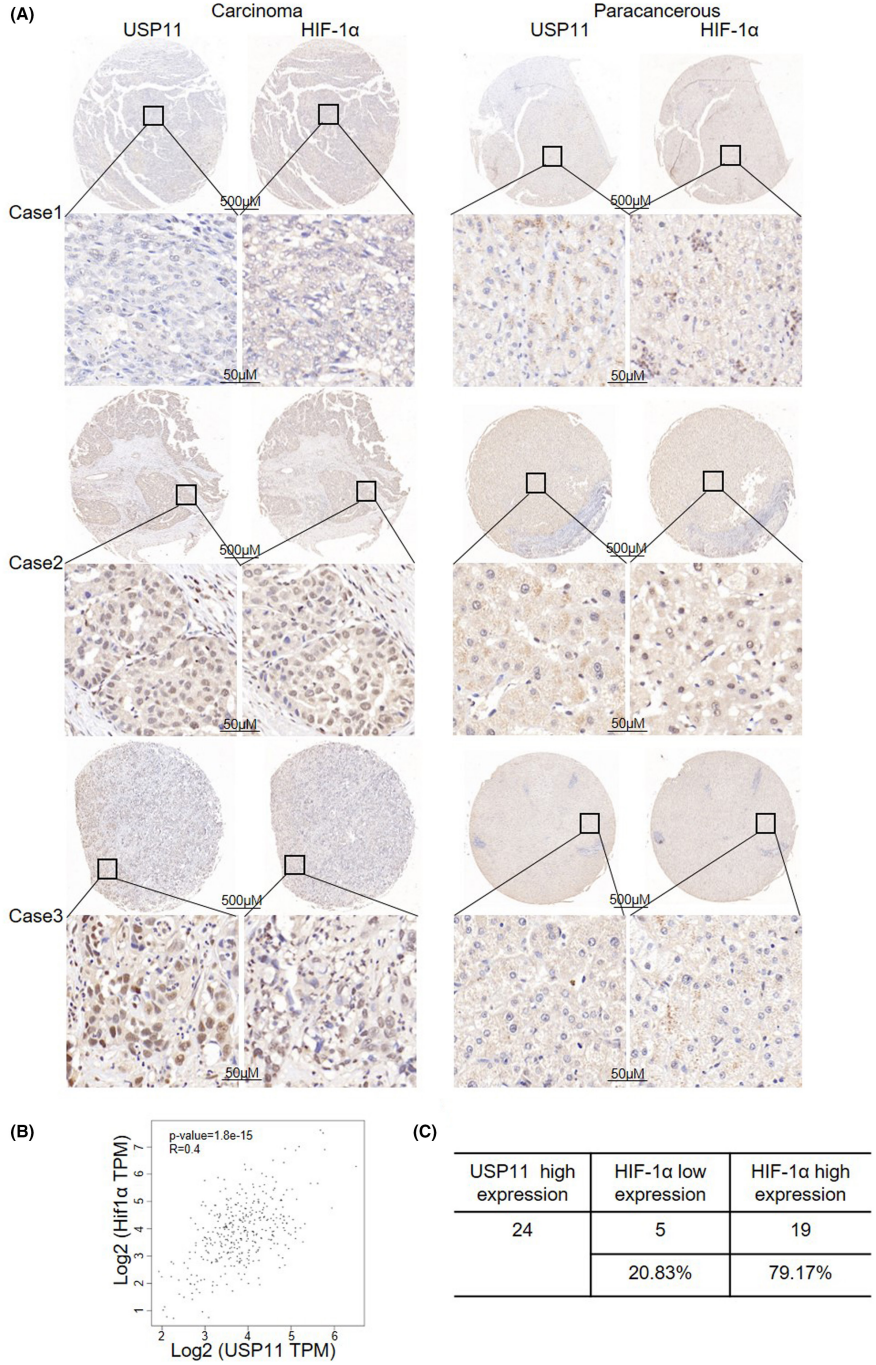
Analysis of the correlation between USP11 and HIF‐1α in clinical samples. (A) Representative results of the immunohistochemical staining for USP11 and HIF‐1α expression in clinical samples. HIF‐1α expression levels were increased in HCC tissues with high USP11 expression levels. (B) Analysis the correlation between USP11 and HIF‐1α in GEO databases. (C) In the USP11 high‐expression group, high HIF‐1α expression was found in 62.5% (15/24) and low HIF‐1α expression was found in 37.5% (9/24) of the patients.

Previous studies showed that highly expression of USP11 in HCC was associated with poorer clinical outcomes.^16^ Moreover, a poor prognosis was related to high HIF‐1α expression in HCC.^7^, ^16^ We examined the relationship between USP11 and HIF‐1α using the GEPIA (TCGA) database and found a positive association between USP11 and HIF‐1α in HCC (*p* < 0.05) (Figure 5B). On a tissue microarray made up of 29 pairs of malignant liver tissues, immunohistochemistry staining for USP11 and HIF‐1α proteins was done in order to further support these findings. A strong positive association between HIF‐1α and USP11 was found, as illustrated in Figure 5A. In the USP11 high‐expression group, higher HIF‐1α expression was observed in 62.5% (15/24) of the patients and lower HIF‐1α expression was found in 37.5% (9/24) (Figure 5C), further confirming the positive correlation between HIF‐1α and USP11.

## 4 DISCUSSION

**FIGURE 6:**
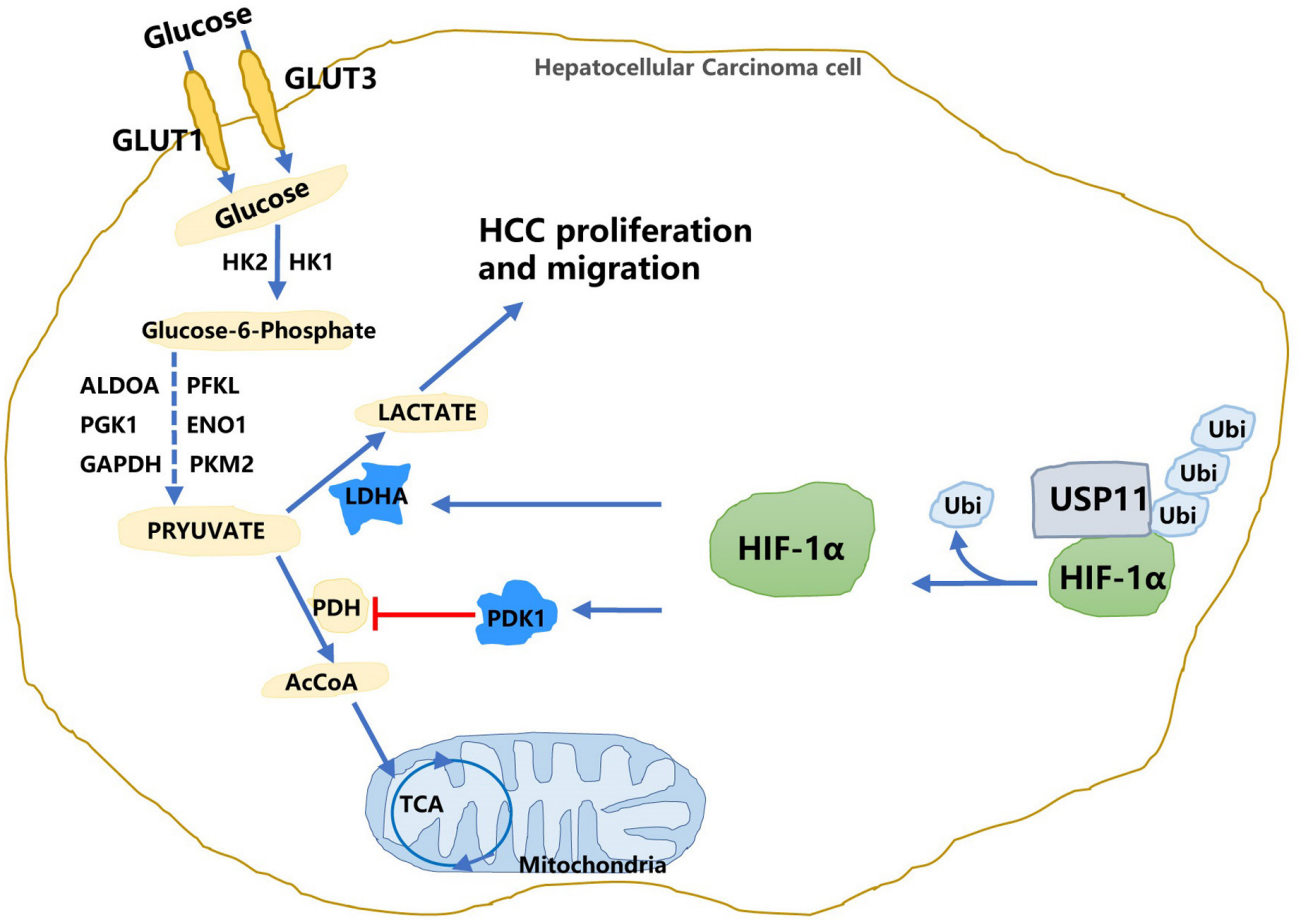
Schematic representation showing how the USP11/HIF‐1α pathway mediated aggressive behaviours in HCC through enhancing glycolysis.

In the present study, we identified USP11 as an upstream regulator of HIF‐1α in HCC cells. We demonstrated that USP11 overexpression promotes HIF‐1α stabilization and enhances glycolysis through PDK1 and LDHA‐related pathways, eventually leading to tumour cell proliferation and metastasis (Figure 6). Abnormal expression of USP11 has been reported in many tumours, including diffuse large B‐cell lymphoma,^33^ colorectal cancer,^34^ gastric cancer^16^ and HCC.^35^ According to previous reports, USP11 promoted HCC cell metastasis and could serve as a promising clinical prognostic marker.^35^ Moreover, USP11 could destabilize Krüppel‐like factor 4 (KLF4), a DNA‐binding transcription factor participated in tumorigenesis and acts as an HCC suppressor, thereby mediating hepatic tumorigenesis.^36^ Combination and deubiquitination of USP11 to nuclear factor 90 (NF90) stabilized the protein expression and promoted HCC cell metastasis and proliferation.^14^ Our previous research uncovered a positive feedback loop between E2F1/USP11 that drives HCC progression and suppresses autophagy by activating the ERK/mTOR signalling pathway.^16^ However, as an important member of the deubiquitinase family, USP11 may regulate more protein activities to influence HCC progression. In this study, we substantiated that USP11 silencing in HCC cells drastically decreased HIF‐1α expression.

One of the prominent features of advanced solid tumours is the insufficient blood supply. In this respect, HCC cells are more likely to experience hypoxia, which promotes cancer cells to alter their metabolic processes and signalling pathways to survival.^37^ HIF‐1α is a well‐defined hypoxia‐responsive factor regulating various biological processes, including angiogenesis, cellular metabolism, drug resistance and proliferation in tumour cells.^38^ In an environment with normal oxygen levels, HIF‐1α is hydroxylated and promptly degraded by the ubiquitin‐proteasome system. In contrast, in hypoxic conditions, HIF‐1α translocates into the nucleus to stimulate downstream gene transcription.^39^ HIF‐1α protein expression was comparable to the USP11 protein and represents a prospective target of the ubiquitin‐proteasome system. Besides, overexpression of USP11 did not influence HIF‐1α at the mRNA level, indicating that exploration of the regulatory mechanism of HIF‐1α by USP11 should focus on the post‐translational level. The Co‐IP assay proved that USP11 could combine with the HIF‐1α protein complex in HCC cells. A previous study demonstrated that a combination of USP11 catalyses the deubiquitination of IκBα and activates the NF‐κB pathway.^40^ Another study showed that USP11 deubiquitinates and stabilizes promyelocytic leukaemia protein, thereby counteracting the functions of ubiquitin ligases and controlling Notch‐induced malignancy in brain tumours.^41^ We found that USP11 could induce HIF‐1α deubiquitination and stabilization. The functional influence of USP11 and HIF‐1α on HCC cells was further investigated. The results indicated that the proliferation, migratory and colony formation ability of HCC cells increased after USP11 overexpression in both NC and siHIF‐1α transfected groups, suggesting that HIF‐1α acts as the functional downstream target of USP11.

Aerobic glycolysis is a common feature of cancer cell glucose metabolism, where glucose is mainly converted into lactate.^42^ Aerobic glycolysis enhances cancer cell survival under extreme conditions by increasing biosynthesis, suppressing apoptosis and generating signalling metabolites.^43^ It is well established that the oxygen‐sensing transcription factor HIF‐1 controls whether glucose is metabolized by glycolysis or oxidation. HIF‐1α is quickly hydroxylated and undergoes ubiquitin‐mediated degradation under physiological conditions. This process can be stopped by a number of stimuli in the tumour microenvironment, which causes the production of the HIF‐1α protein to increase the rate of aerobic glycolysis in tumour cells.^43^ Fanny Dupuy et al.^44^ demonstrated that higher expression of HIF‐1α in liver‐metastatic breast cancer cells targets PDK1 and regulates cell metabolism and metastasis.

Among all genes enriched in glycolysis pathways after USP11 knockdown, the fold change of LDHA is the most significant. As another key target of HIF‐1α, LDHA catalyses the reduction inf pyruvate to lactate and maintains cell viability in hypoxic situations by making up for the decreased activity of oxidative mitochondrial.^45^ Herein, we further confirmed that the knockdown of USP11 reduced HIF‐1α activity through PDK1 and LDHA pathways. Overexpression of USP11 enhances HIF‐1α as well as PDK1 and LDHA protein. The lactic acid content induced by glycolysis was reduced after siUSP11 transfection. Moreover, HIF‐1α overexpression rescued the decreased lactic acid levels after siUSP11 transfection in HCC cells.

As far as we are aware, this study is the first to identify HIF‐1α as a USP11 downstream target. The deubiquitination of HIF‐1α by USP11 stabilized the HIF‐1α protein and promoted HCC cell glycolysis through LDHA and PDK1 pathways. As a result, the proliferation, migration and colony formation ability of HCC cells were enhanced. When viewed collectively, our findings corroborate that USP11 has huge prospects for application as a prognostic biomarker and a therapeutic target for the treatment of HCC.

## AUTHOR CONTRIBUTIONS

**Lijun Qiao:** Conceptualization (supporting); data curation (lead); funding acquisition (equal); investigation (equal); writing – original draft (equal). **Weibin Hu:** Investigation (supporting); methodology (supporting). **Linzhi Li:** Investigation (supporting); methodology (supporting). **Xin Chen:** Methodology (supporting). **Liping Liu:** Conceptualization (equal); project administration (equal); supervision (equal). **Jingbo Wang:** Conceptualization (equal); funding acquisition (equal); project administration (equal); supervision (equal); validation (equal); writing – original draft (equal); writing – review and editing (equal).

## FUNDING INFORMATION

This work was supported by the National Natural Science Foundation of China (81903013) and the Youth Innovative Talents Project of Guangdong Education Department (2021KQNCX082).

## CONFLICT OF INTEREST STATEMENT

The authors declare that they have no competing interests.

## INFORMED CONSENT

Written informed consent was obtained from all participants.

## Supporting information


Table S1

Table S2

Table S3

## Data Availability

The RNA sequencing datasets used and/or analysed during the current study are available from https://www.ncbi.nlm.nih.gov/bioproject/PRJNA894454 and Supplemental files. The non‐sequencing data are available from the corresponding author upon reasonable request.
